# GbSOBIR1 confers *Verticillium* wilt resistance by phosphorylating the transcriptional factor GbbHLH171 in *Gossypium barbadense*

**DOI:** 10.1111/pbi.12954

**Published:** 2018-07-24

**Authors:** Yi Zhou, Longqing Sun, Ghulam Mustafa Wassan, Xin He, Muhammad Shaban, Lin Zhang, Longfu Zhu, Xianlong Zhang

**Affiliations:** ^1^ National Key Laboratory of Crop Genetic Improvement Huazhong Agricultural University Wuhan Hubei China

**Keywords:** *Gossypium barbadense*, *Verticillium* wilt, GbSOBIR1, GbbHLH171

## Abstract

Receptor‐like kinases (RLKs) are important components of plant innate immunity. Although recent studies have revealed that the RLK suppressor of BIR1‐1 (SOBIR1) can interact with multiple receptor‐like proteins and is required for resistance against fungal pathogens, how the signal is transduced and triggers immune responses remains enigmatic. In this study, we identified a defence‐related RLK from *Gossypium barbadense* (designated GbSOBIR1) and investigated its functional mechanism. Expression of the *GbSOBIR1* gene is ubiquitous in cotton plants and is induced by *Verticillium dahliae* inoculation. Knock‐down of *GbSOBIR1* by virus‐induced gene silencing resulted in attenuated resistance of cotton plants to *V. dahliae*, while heterologous overexpression of *GbSOBIR1* in *Arabidopsis* improves resistance. We also found that the kinase region of GbSOBIR1 interacts with a basic helix‐loop‐helix (bHLH) transcription factor identified as GbbHLH171 in a yeast‐two‐hybrid screen. GbbHLH171 could interact with and be phosphorylated by GbSOBIR1 *in vitro* and *in vivo* and contributes positively to the resistance of cotton against *V. dahliae*. Furthermore, we found that this phosphorylation is essential to the transcriptional activity and functional role of GbbHLH171. We also show by spectrometric analysis and site‐directed mutagenesis that Ser413 is the GbSOBIR1‐mediated phosphorylation site of GbbHLH171. These results demonstrate that GbSOBIR1 interacts with GbbHLH171 and plays a critical role in cotton resistance to *V. dahliae*.

## Introduction

*Verticillium* wilt, mainly caused by the soil‐borne fungus *Verticillium dahliae*, is a devastating disease affecting cotton production all over the world. There is no efficient chemical pesticide available for cotton *Verticillium* wilt, and there are few germplasms of upland cotton that are immune or highly resistant to *V. dahliae* (Aguado *et al*., 2008; Zhang *et al*., 2012b). Nevertheless, progress has been made towards understanding the molecular mechanisms of disease tolerance in cotton. Studies using methods such as RNA‐Seq and proteomic analysis reveal that genes involved in lignin biosynthesis, salicylic acid (SA), jasmonic acid (JA) and brassinosteroid (BR) signalling pathways play important roles in cotton resistance to *V. dahliae* (Gao *et al*., 2013a; Wang *et al*., 2011; Xu *et al*., 2011). Other genes that may participate in the resistance mechanism include *Gbvdr5*,* GhBAK1*,* GhSSN*,* GbWRKY1* and *GbERF1‐like* (Gao *et al*., 2013b; Guo *et al*., 2016; Li *et al*., 2014; Sun *et al*., 2014; Yang *et al*., 2015).

Until now, *Ve1* from tomato (*Solanum lycopersicum*) has been the only major resistance gene to *Verticillium* wilt and was identified through map‐based cloning (Kawchuk *et al*., 2001). *Ve1* encodes a leucine‐rich repeat receptor‐like protein (LRR‐RLP) cell surface receptor with extracellular LRR domains but lacks a cytoplasmic signalling domain and mediates resistance against race 1 strains of *V. dahliae* and *V. albo‐atrum* (Fradin *et al*., 2009; Wang *et al*., 2010). Interfamily transfer of tomato *Ve1* mediates *Verticillium* resistance in Arabidopsis, suggesting that the signalling cascade exploited by Ve1 may be conserved in different plant families (Fradin *et al*., 2011). *Ve1* is activated by Ave1, an effector of *Verticillium* race 1 strains, and induces a hypersensitive response (HR) in tobacco leaves (de Jonge *et al*., 2012). It also confers resistance to race 1 *V. dahliae* when expressed in tobacco and cotton (Song *et al*., 2018).

Suppressor of BIR1‐1 (SOBIR1) is a receptor‐like kinase (RLK) initially identified as a suppressor of BIR1 (BAK1‐interacting receptor‐like kinase 1) and plays a positive role in *Arabidopsis* immunity (Gao *et al*., 2009). SOBIR1 is involved in plant immunity through interaction with a series of RLPs in tomato (Gust and Felix, 2014; Liebrand *et al*., 2013, 2014) and is also required for the function of *Arabidopsis* RLP1, RLP23, RLP30 and RLP42 in the recognition of pathogen effectors (Albert *et al*., 2015; Jehle *et al*., 2013; Zhang *et al*., 2013, 2014). SOBIR1 can interact with BAK1 in the absence of BIR1 to mediate cell death and *defence* responses (Liu *et al*., 2016) and is necessary for tomato *I*‐gene‐mediated *Fusarium wilt* resistance (Catanzariti *et al*., 2017). SOBIR1 is also required for Ve1‐mediated HR in tobacco and the resistance of *Arabidopsis* against the fungal pathogen *V. dahliae* (Liebrand *et al*., 2013). However, the downstream signalling pathways mediated by SOBIR1 are poorly understood.

bHLH proteins are the second largest class of plant transcription factors, and they play essential roles in a wide range of physiological and developmental processes (Chinnusamy *et al*., 2003; Reyes‐Olalde *et al*., 2017; Schaart *et al*., 2013; Yao *et al*., 2017). They are also involved in *defence* responses in plants. The bHLH transcription factor MYC2 is considered to be a central factor in JA signalling in Arabidopsis and increased resistance against necrotrophic pathogens such as *Botrytis cinerea* and *Plectosphaerella cucumerina* can be observed in the *myc2* mutant, due to elevated JA‐dependent *defence*s (Lorenzo *et al*., 2004). Another Arabidopsis bHLH transcription factor, ILR3, can interact directly with the coat protein of alfalfa mosaic virus (AMV) and regulate *defence* responses (Aparicio and Pallas, 2017). Furthermore, Arabidopsis bHLH84 interacts with SNC1 and RPS4, two nucleotide‐binding and leucine‐rich repeat domain‐containing (NB‐LRR) immune receptors, and confers immunity against *Pseudomonas syringae* (Xu *et al*., 2014).

In this report, we identify an RLK‐encoding gene in cotton, which shows high homology with SOBIR1 in *Arabidopsis* and therefore is designated GbSOBIR1. We show that expression of GbSOBIR1 is induced by *V. dahliae* inoculation and that down‐regulation of its expression attenuates plant resistance to pathogen infection. Importantly, we find that GbSOBIR1 can interact with and phosphorylate GbbHLH171, a bHLH transcription factor in cotton. This phosphorylation is essential for the transcriptional activity and functional role of GbbHLH171. Our results provide a critical line of evidence showing that GbSOBIR1 could activate the transcriptional activity of GbbHLH171, and synergistic actions of the two proteins are important for resistance against *V. dahliae* in cotton plants.

## Results

### Identification of the *GbSOBIR1* gene and its expression profile

**Figure 1:**
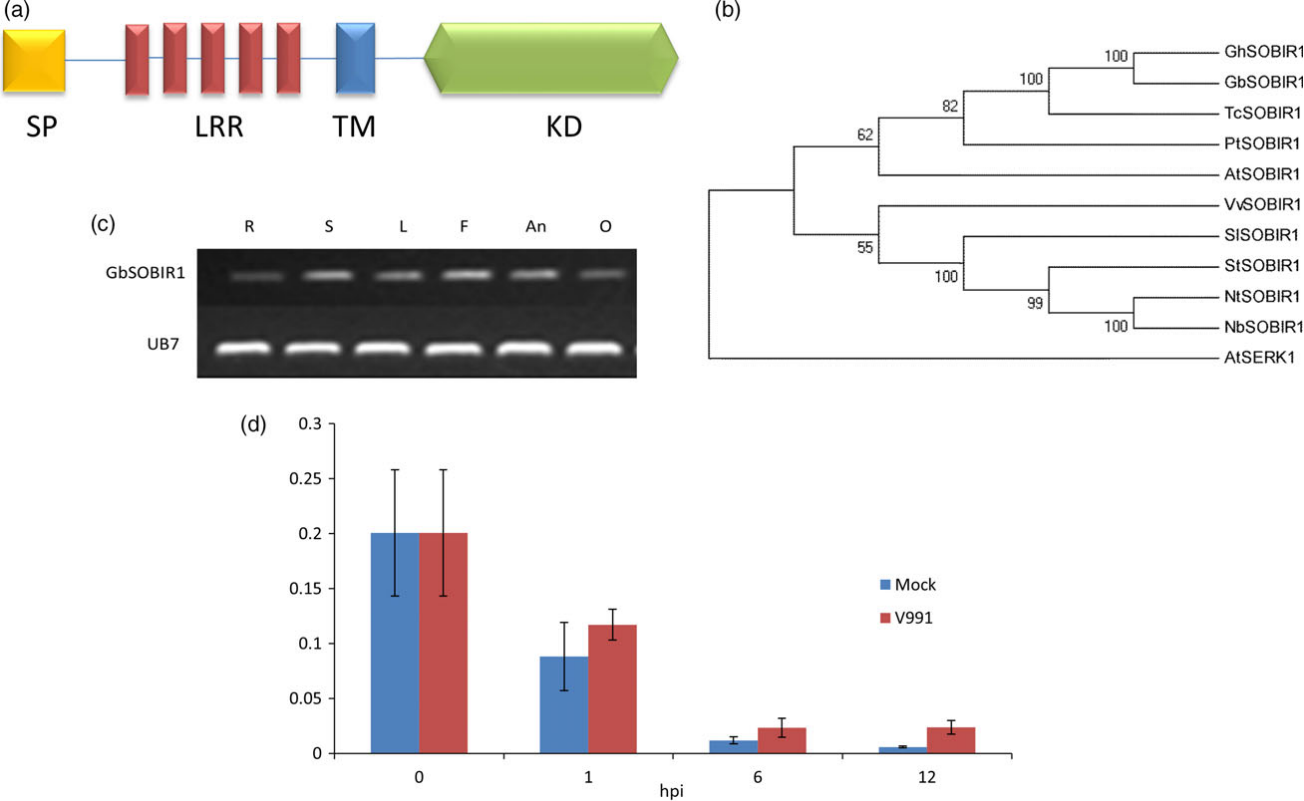
Structural, phylogenetic and expression analysis of GbSOBIR1. (a) Structural model of GbSOBIR1 protein, SP: signal peptide, LRRs: leucine‐rich repeat domains, TM: transmembrane domain, KD: kinase domain. (b) Phylogeny of the SOBIR1 proteins from Gossypium hirsutum (Gh), Gossypium barbadense (Gb), Theobroma cacao (Tc), Populus trichocarpa (Pt), Arabidopsis thaliana (At), Vitis vinifera (Vv), Solanum lycopersicum (Sl), Solanum tuberosum (St), Nicotiana tabacum (Nt) and Nicotiana benthamiana (Nb). The tree was constructed using the neighbor‐joining algorithm in MEGA 5 with 1000 bootstrap replications (http://www.megasoftware.net/). (c) RT‐PCR analysis of GbSOBI1 expression in different tissues. Total RNA was isolated from roots (R), stems (S), leaves (L), flower (F), anther (An) and ovule (O) of the Gossypium barbadense cv 7124. The UB7 gene was used as a control. (d) qRT–PCR analysis of GbSOBIR1 expression in cotton roots inoculated with *V. dahliae*. Total RNAs were extracted from roots of 14 day‐old seedlings at 0–12 h after inoculation. The experiments were repeated three times with similar results. The values are the means ± SD for three technical replicates. The transcript levels of each gene were normalized to UB7. hpi, hours post inoculation.

We previously characterized the eukaryotic P450 gene *SSN* (*SILENCING‐INDUCED STEM NECROSIS*); a lesion mimic phenotype was observed in knock‐down mutant *ssn* lines (Sun *et al*., 2014). A set of genes were identified that are differentially expressed in roots between the knock‐down mutant (Ri15) and WT seedlings. Among them, one receptor‐like kinase gene was further studied, and we identified the homolog of this gene in the cotton cultivar *G. barbadense cv* 7124. This is found to encode an RLK that contains five LRR domains (Figure 1a). Phylogenetic analysis of this cotton RLK and several RLKs from other plants showed that it was evolutionarily close to the *Arabidopsis* SOBIR1 (Figure 1b), which plays important roles in plant *defence* responses. Amino acid sequence alignment of GbSOBIR1 and previously reported SlSOBIR1, AtSOBIR1, NbSOBIR1 shows that each pair was highly homologous (more than 60% identical) (Figure S1). Based on the phylogenetic results, this gene was designated *GbSOBIR1*.

The expression of *GbSOBIR1* in various tissues of the cotton plant was examined by RT‐PCR. As shown in Figure 1c, the gene was expressed in all tissues investigated. We also checked *V. dahliae*‐induced expression of *GbSOBIR1* by qRT‐PCR analysis, and it was found to be slightly induced during early stages of *V. dahliae* infection (Figure 1d). To further investigate the involvement of GbSOBIR1 in disease responses, gene expression was analysed after exogenous treatment with the *defence*‐related phytohormones SA and JA. As shown in Figure S2, expression of *GbSOBIR1* was enhanced slightly by exogenous application of SA but was down‐regulated following JA treatment. These results suggest that GbSOBIR1 may be involved in *defence* against *V. dahliae* infection in the cotton plant.

### Reduced *GbSOBIR1* expression results in increased susceptibility of cotton to *V. dahliae* infection

**Figure 2:**
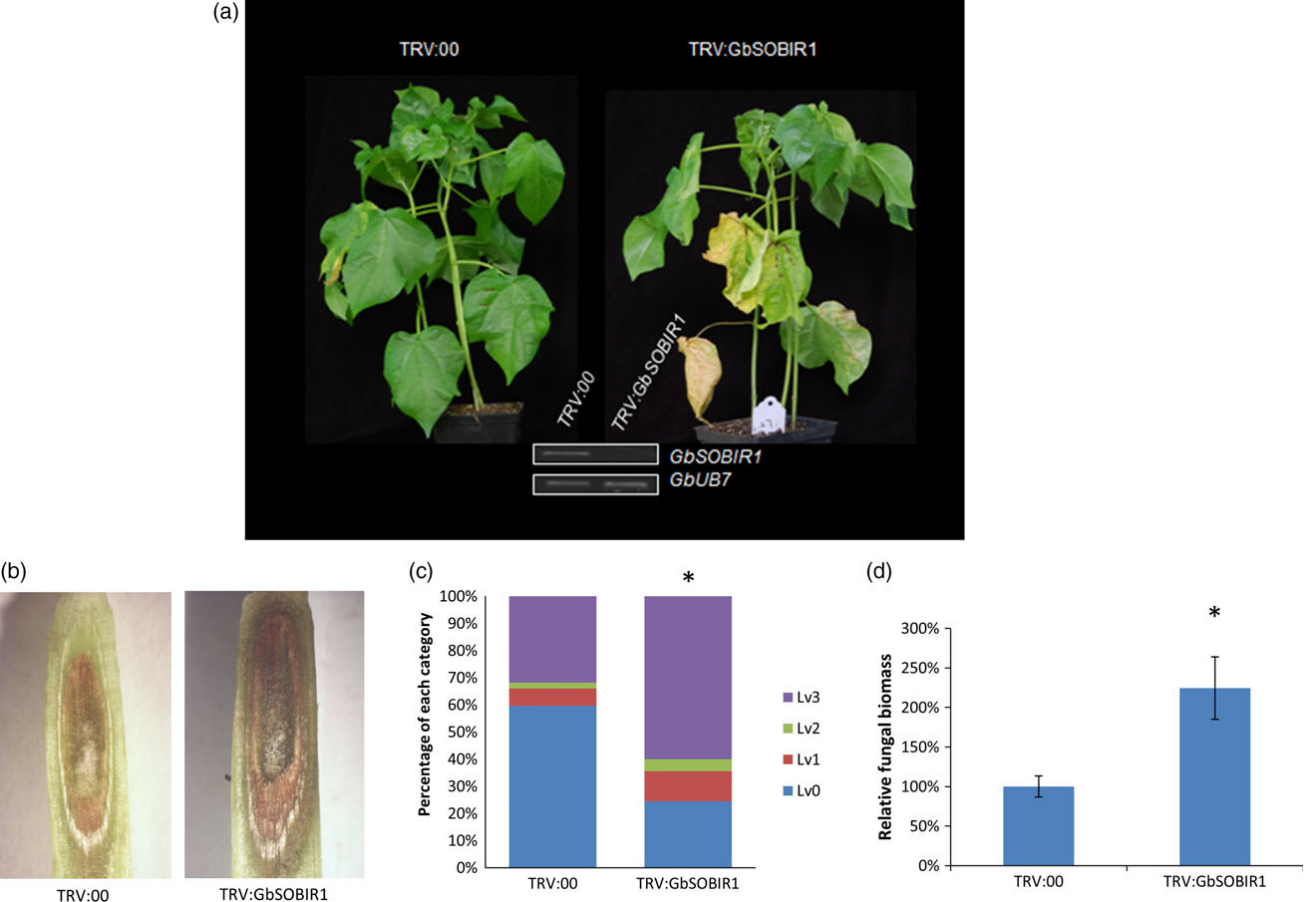
Increased susceptibility of *GbSOBIR1*‐silenced cotton plants to *V. dahliae*. (a) Disease symptoms of GbSOBIR1‐silenced plants infected by *V. dahliae* strain V991. Seedlings were inoculated with *V. dahliae* 2 weeks after VIGS and photos were taken 14 days after inoculation. The successful knock‐down of GbSOBIR1 was confirmed through RT‐PCR analysis. (b) Darkened vascular tissues of control (TRV:00) and GbSOBIR1‐silenced (TRV: GbSOBIR1) cotton plants tissues after *V. dahliae* inoculation (c) Leaves of Inoculated plants were classified according to the disease symptoms. Lv0: healthy leaves, Lv1: leaves showing some necrosis or chlorosis, Lv2: leaves showing severe necrosis or chlorosis, Lv3: dead/shed leaves. Asterisk (*) indicates *P* < 0.05 by Wilcoxon rank‐sum test. The experiments were repeated three times with similar results and at least 15 plants of each line were used each time. (d) Fungal biomass determined by quantitative real‐time PCR in cotton plants 14 days after inoculation. The values are the means ± SD, statistical analyses were performed using Student's *t* test. All of the experiments were repeated at least three times with similar results.

To elucidate the role of GbSOBIR1 during cotton *defence* to *V. dahliae*, tobacco rattle virus (TRV)‐based virus‐induced gene silencing (VIGS) was employed to knock down the transcript of *GbSOBIR1*. Cotton seedlings were grown for 10 days, after which they were treated with either an empty recombinant TRV vector (*TRV:00*) or a TRV vector targeting *GbSOBIR1* (*TRV: GbSOBIR1*). Two weeks after treatment, the reduced expression of the *GbSOBIR1* gene was confirmed through RT‐PCR analysis (Figure 2a). The plants were then inoculated with the *V. dahliae* strain V991. Control plants displayed typical disease symptoms such as wilted leaves and darkened vascular bundles. However, the *GbSOBIR1*‐silenced plants were more severely affected than the control plants (Figure 2a,b); 24% and 60% of the leaves from *TRV:GbSOBIR1* and *TRV:00* plants, respectively, showed no necrosis or chlorosis, whereas 60% and 32% of the leaves from *TRV:GbSOBIR1* and *TRV:00* plants, respectively, were totally necrotic or shed (Figure 2c). The phenotypes correlated with the degree of *Verticillium* colonization, as determined by a fungal biomass analysis (Figure 2d). These results show that knock‐down of the *GbSOBIR1* gene attenuates the resistance of cotton plants to *V. dahliae* infection.

### Overexpression of *GbSOBIR1* in transgenic *Arabidopsis* plants enhances *V. dahliae* resistance

**Figure 3:**
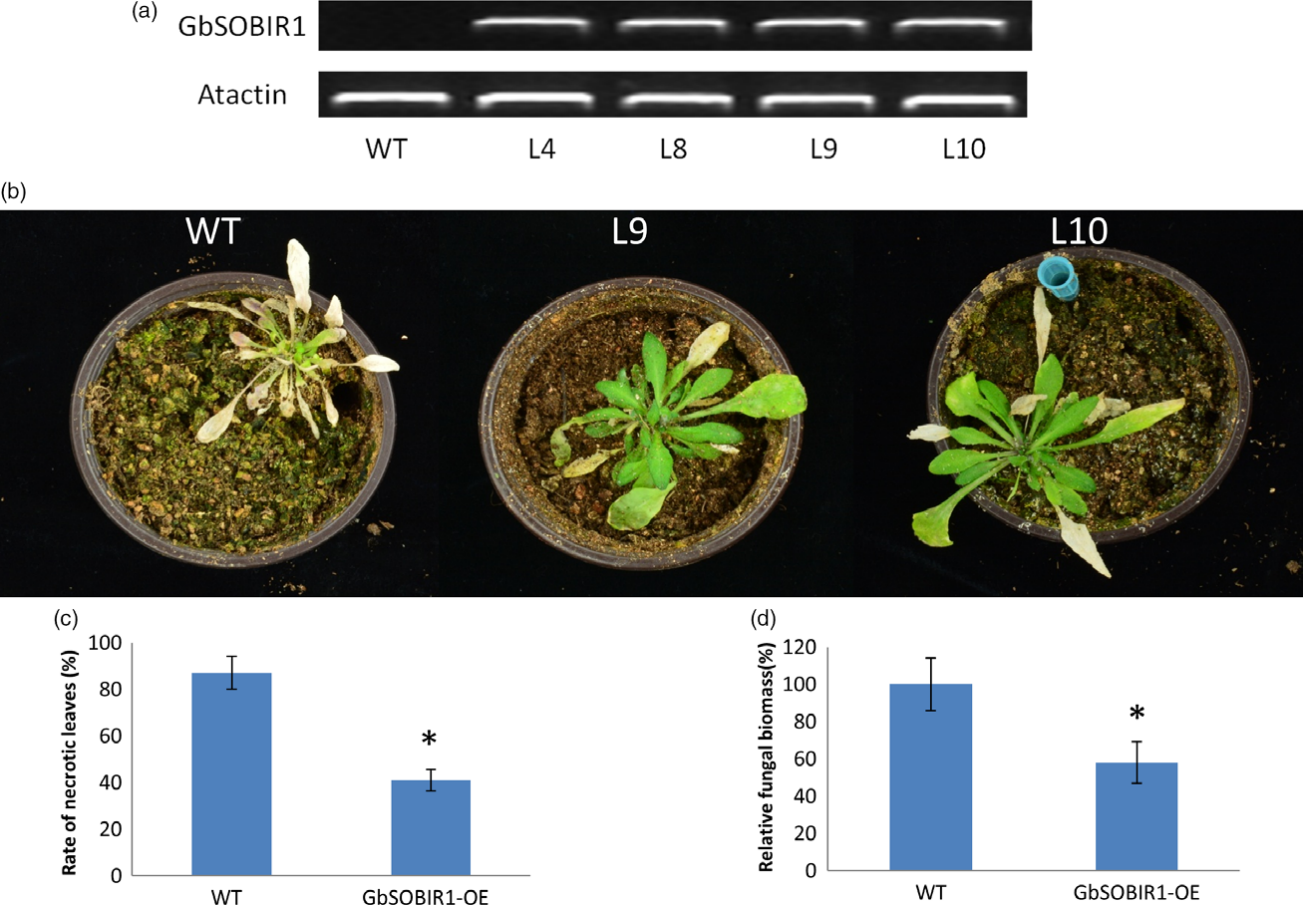
Enhanced *V. dahliae* resistance of the *Arabidopsis* plants overexpressing *GbSOBIR1*. (a) Expression levels of GbSOBIR1 driven by the 35S promoter in transgenic Arabidopsis lines (L4, L8, L9 and L10). Actin was used as an internal control. (b) Symptoms of wild‐type and GbSOBIR1 transgenic Arabidopsis plants inoculated with *V. dahliae*. 2‐ to 3‐week‐old Arabidopsis plants were inoculated with *V. dahliae* and re‐planted in soil, with at least 20 plants for each line, and photographed 21 days after inoculation. (c) Rate of necrotic leaves (%) in wild‐type and transgenic Arabidopsis plants. (d) Fungal biomass determined by quantitative real‐time PCR in wild‐type and transgenic Arabidopsis plants. The values are the means ± SD, statistical analyses were performed using Student's *t* test (*P < 0.05). All of the experiments were repeated at least three times with similar results. WT, wild‐type.

An overexpression strategy was also employed to assess the function of the *GbSOBIR1* gene. Unfortunately, we were unable to obtain transgenic plants with RNAi or overexpression of *GbSOBIR1* due to abnormal development or lethality during the plant tissue culture process necessary for cotton transformation. Instead, we generated *Arabidopsis* lines that heterologously expressed *GbSOBIR1* and more than 20 independent transgenic lines were obtained. Four lines with high expression levels of *GbSOBIR1* (Figure 3a) were chosen for further analysis. Wild‐type and transgenic plants were inoculated with *V. dahliae* strain V991, and disease development was monitored up to 21 days after inoculation. A more resistant phenotype could be observed in the transgenic plants with less stunting, wilting, anthocyanin accumulation, chlorosis, early senescence and necrosis (Figure 3b,c); this was confirmed by fungal biomass analysis (Figure 3d). These results support the view that GbSOBIR1 contributes positively to plant resistance to *V. dahliae*.

### GbSOBIR1 interacts with GbbHLH171 *in vitro* and *in vivo*

**Figure 4:**
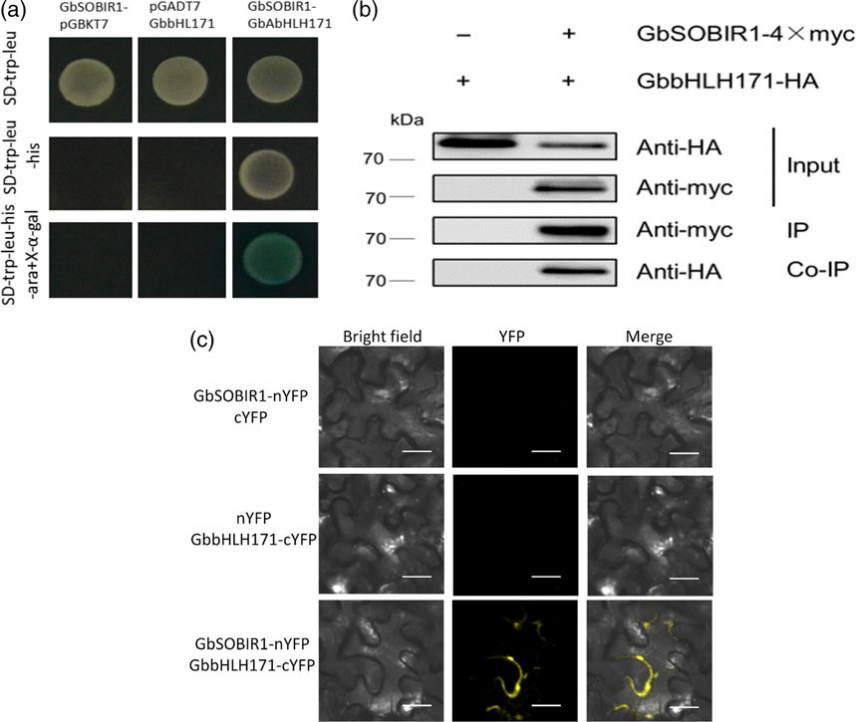
GbSOBIR1 interacts with GbbHLH171 *in vitro* and *in vivo*. (a) GbbHLH171 interacts with GbSOBIR1 in Y2H assays. The empty pGADT7 and pGBKT7 were used as negative controls. Blue colonies on SD‐Trp‐Leu‐His + X‐α‐gal medium indicated positive interactions. (b) Coimmunoprecipitation (Co‐IP) of GbSOBIR1 and GbbHLH171. Total proteins expressed in *N. benthamiana* leaves were subjected to immunoprecipitation (IP) with anti‐Myc beads, followed by immunoblot analysis with anti‐Myc antibodies to detect GbSOBIR1‐Myc as well as with anti‐HA antibodies to detect GbbHLH171‐HA. (c) Bimolecular fluorescence complementation assay to detect the interactions of GbbHLH171 and GbSOBIR1 at the plasma membrane. The GbSOBIR1‐nYFP + cYFP and nYPF + GbbHLH171‐cYFP construct pairs were used as negative control. YFP fluorescence was detected 2 days after infiltration. Bars, 20 μm.

To understand the regulatory network by which GbSOBIR1 confers resistance to *V. dahliae*, the kinase region of GbSOBIR1 was cloned into the pGBKT7 vector to construct a bait plasmid encoding a fusion protein with the DNA‐binding domain of GAL4. The fusion plasmid was used to screen a *V. dahliae*‐inoculated cotton root library using the yeast‐two‐hybrid (Y2H) system, and one bHLH transcript factor was identified as an interacting protein of GbSOBIR1. The cDNA encoding this protein is 2697 bp in length with an open reading frame of 1941 bp, encoding a protein that contains 646 amino acid residues. A bHLH and R2R3‐MYB transcription factor N‐terminal domain and a helix‐loop‐helix domain can be found in the predicted protein. Based on a previous phylogenetic analysis (Yan *et al*., 2015), we named this protein GbbHLH171 (He *et al*., 2018). The ORF of *GbbHLH171* was cloned and mated to GbSOBIR1 in yeast to confirm the interaction (Figure 4a). To verify the interaction *in vivo*, GbSOBIR1 fused to the Myc epitope tag and GbbHLH171 fused to the HA epitope tag were generated and transiently co‐expressed in *N. benthamiana* leaves to perform co‐immunoprecipitation experiments. HA‐tagged GbbHLH171 was co‐immunopurified with GbSOBIR1 when Myc‐tagged GbSOBIR1 was used to capture the GbbHLH171 complex (Figure 4b).

How GbbHLH171, a transcription factor, interacts with membrane‐located GbSOBIR1 needs to be explored. Another bHLH transcriptional factor, AaMYC2, has been found to form complexes with several proteins in the plasma membrane (Shen *et al*., 2016). To test whether GbbHLH171 interacts similarly with GbSOBIR1, bimolecular fluorescence complementation (BiFC) assays were carried out to assess the interaction between GbSOBIR1 and GbbHLH171 in plant cells. When GbSOBIR1‐nYFP and GbbHLH171‐cYFP were co‐expressed in *N. benthamiana*, YFP fluorescence was detected at the plasma membrane, while no fluorescence was detected when GbSOBIR1‐nYFP and cYFP or nYFP and GbbHLH171‐cYFP were co‐expressed (Figure 4c). These results indicate that GbSOBIR1 interacts with GbbHLH171 both *in vitro* and *in vivo*, and the subcellular location of this interaction is at the plasma membrane.

### GbbHLH171 plays a positive role in cotton resistance to *V. dahliae*

**Figure 5:**
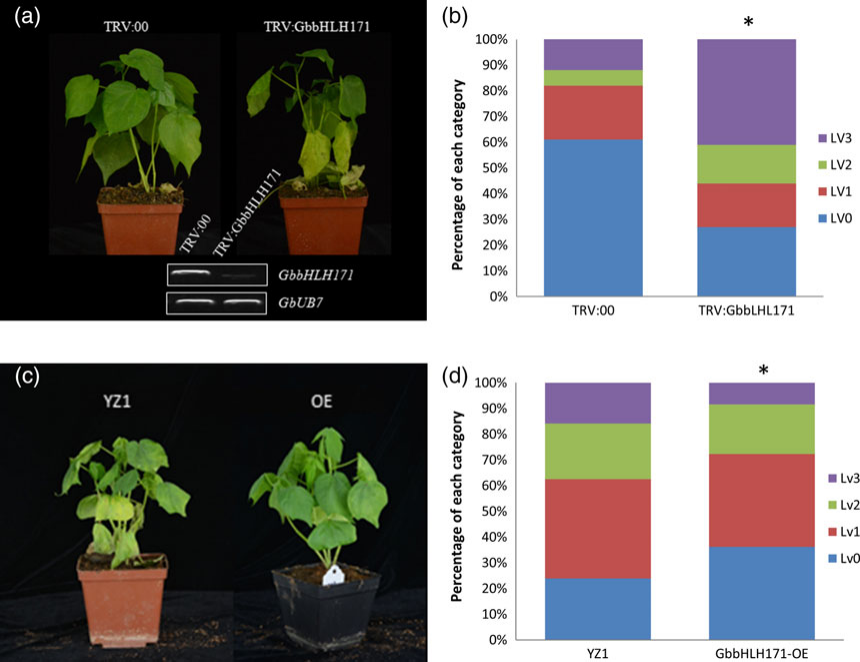
GbbHLH171 is a positive regulator in cotton resistance against *V. dahliae*. (a, c) Disease symptom of GbbHLH171‐silenced (a) and ‐overexpressing (c) plants infected by *V. dahliae* strain V991. The experiments were repeated three times with similar results and at least 15 plants of each line were used each time. (b, d) Leaves of GbbHLH171‐silenced (b) and ‐overexpressing (d) plants were classified according to the disease symptoms. Asterisk (*) indicates *P* < 0.05 by Wilcoxon rank‐sum test.

To determine whether GbbHLH171 is involved in resistance against *V. dahliae*, we first examined its expression pattern. As shown in Figure S3A, *GbbHLH171* is predominantly expressed in cotton roots and is induced within 1 h after inoculation with *V. dahliae* (Figure S3B). VIGS was employed to knock down *GbbHLH171* expression. TRV constructs targeting *GbbHLH171* were generated, and cotton plants were infiltrated with both *TRV:GbbHLH171* and the negative control *TRV:00*. Two weeks after TRV infiltration, plants were inoculated with the *V. dahliae* strain V991 and subsequently monitored for the development of disease symptoms. Knock‐down of *GbbHLH171* resulted in more severe disease symptoms compared with the *TRV:00* plants (Figure 5a,b). We also obtained *GbbHLH171* overexpression cotton lines. When challenged with V991, plants overexpressing *GbbHLH171* displayed a more resistant phenotype with less leaves showing chlorosis or necrosis (Figure 5c,d). These results indicate that GbbHLH171 is required for resistance to *V. dahliae* in cotton.

### Phosphorylation of GbbHLH171 by GbSOBIR1 is required for its functional role

**Figure 6:**
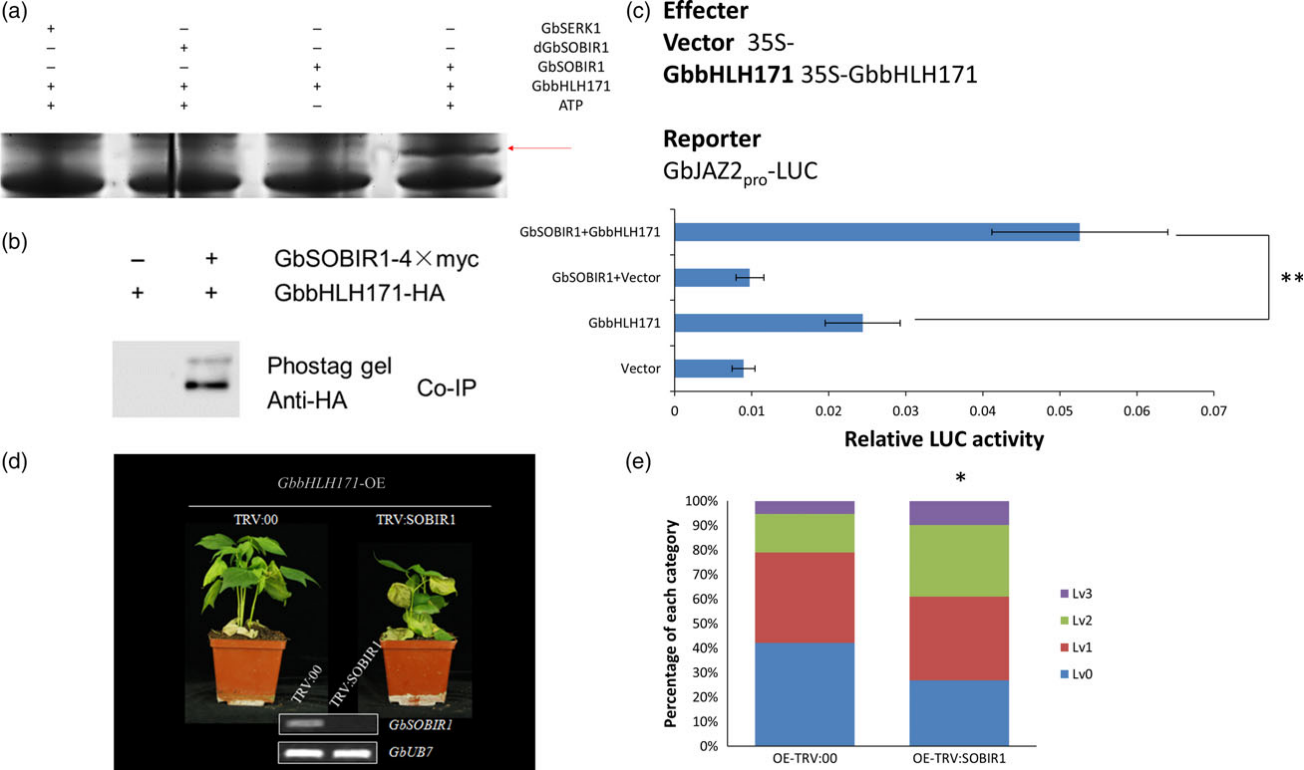
GbSOBIR1‐mediated phosphorylation of GbbHLH171 is required for defense. (a) Phosphorylation of GbbHLH171 by GbSOBIR1 was verified by in vitro phosphorylation assays using Phostag SDS‐PAGE. dGbSOBIR1(denatured GbSOBIR1) and GbSERK1 was used as negative controls. Red arrows indicate positive phosphorylation. (b) Immunoprecipitates from Figure 4b were subjected to SDS‐PAGE containing Phos‐tag and MnCl2 followed by western blot using anti‐HA antibodies. (c) LUC signal intensity of *N. benthamiana* leaves 48 h after co‐infiltration with the same amount of Agrobacterium cells harboring different constructs. The values are the means ± SD, statistical analyses were performed using Student's *t* test (*P < 0.05). The experiments were repeated three times with similar results. (d) Disease symptom of GbbHLH171 overexpressing plants after VIGS. Agrobacterium cells containing TRV:00 or TRV:GbSOBIR1 vectors were injected into cotyledons of GbbHLH171 overexpressing plants followed by *V. dahliae* inoculation 2 weeks later. The experiments were repeated three times with similar results and at least 15 plants of each line were used each time. (e) Classification of leaves according to the disease symptoms in (d). Asterisk (*) indicates *P* < 0.05 by Wilcoxon rank‐sum test.

To test whether GbbHLH171 is a substrate for phosphorylation by GbSOBIR1, proteins of GbSOBIR1 and GbbHLH171 were obtained through high‐yield wheat germ cell‐free and prokaryotic protein expression systems, respectively. An *in vitro* phosphorylation assay was carried out and visualized by SDS‐PAGE containing Phos‐tag and MnCl_2_. As shown in Figure 6a, GbbHLH171 was phosphorylated by GbSOBIR1. GbSERK1 protein was used as a negative control. Furthermore, the immunopurified proteins from the co‐immunoprecipitation experiments were also subject to SDS‐PAGE containing Phos‐tag and MnCl_2_ and then visualized by Western blot using anti‐HA antibodies. An additional phosphorylated protein band is observed (Figure 6b). Collectively, these results demonstrate that GbbHLH171 can be phosphorylated by GbSOBIR1 both *in vitro* and *in vivo*.

The phosphorylation of AtMYC2 was previously reported to be important for its function to regulate gene transcription (Zhai *et al*., 2013). As another bHLH transcription factor, it was investigated whether the GbSOBIR1‐mediated phosphorylation similarly affects the transcriptional activity of GbbHLH171 and its role in resistance to *V. dahliae*. As previously reported, the *Arabidopsis JAZ2* promoter contains G‐boxes that are sufficient for activation by MYC bHLH transcription factors (Figueroa and Browse, 2012). Because there are G‐boxes in the promoter region of *GbJAZ2* as well (Figure S4), we chose *GbJAZ2* as a target to assess the roles of GbbHLH171 and GbSOBIR1 using a LUC assay *in vivo*. The promoter sequence of *GbJAZ2* was isolated from cotton genomic DNA and inserted into the vector pGreenII 0800‐LUC with a *Luc* reporter gene. *Agrobacterium* harbouring the plasmids (Figure 6c) was simultaneously injected into *N. benthamiana* leaves, and Luc fluorescence intensity was examined 60 h later. As shown in Figure 6c, the *GbJAZ2* promoter on its own drove Luc expression weakly, while the fluorescence signal increased when it was cotransformed with *GbbHLH171*, implying that GbbHLH171 can enhance the transcription levels of *GbJAZ2*. When agrobacterium cells containing *35S:GbbHLH171*,* 35S:GbSOBIR1* and *GbJAZ2Pro:Luc* were simultaneously injected, the fluorescence intensity of Luc was even higher than that in the cells cotransformed with *GbJAZ2: Luc* and *35S:GbbHLH171*. These results demonstrate that GbSOBIR1 can enhance the transcriptional activity of GbbHLH171 *in vivo*.

To test whether GbSOBIR1‐mediated phosphorylation of GbbHLH171 influences resistance to *V. dahliae* in cotton plants, we performed VIGS experiments on *GbbHLH171*‐overexpression lines. When the cotyledons were fully expanded, *TRV:00‐* and *TRV:GbSOBIR1*‐containing agrobacterium cells were injected into cotyledons of *GbbHLH171*‐overexpressing plants followed by *V. dahliae* inoculation 2 weeks later. As shown in Figure 6d, the enhanced resistance caused by *GbbHLH171* overexpression was attenuated by targeting GbSOBIR1. More wilt and chlorotic leaves could be observed from the *TRV:GbSOBIR1* plants, and the statistic results were consistent with this (Figure 6e).

### GbbHLH171 phosphorylation at Ser413 by GbSOBIR1 is essential for its transcriptional activity

**Figure 7:**
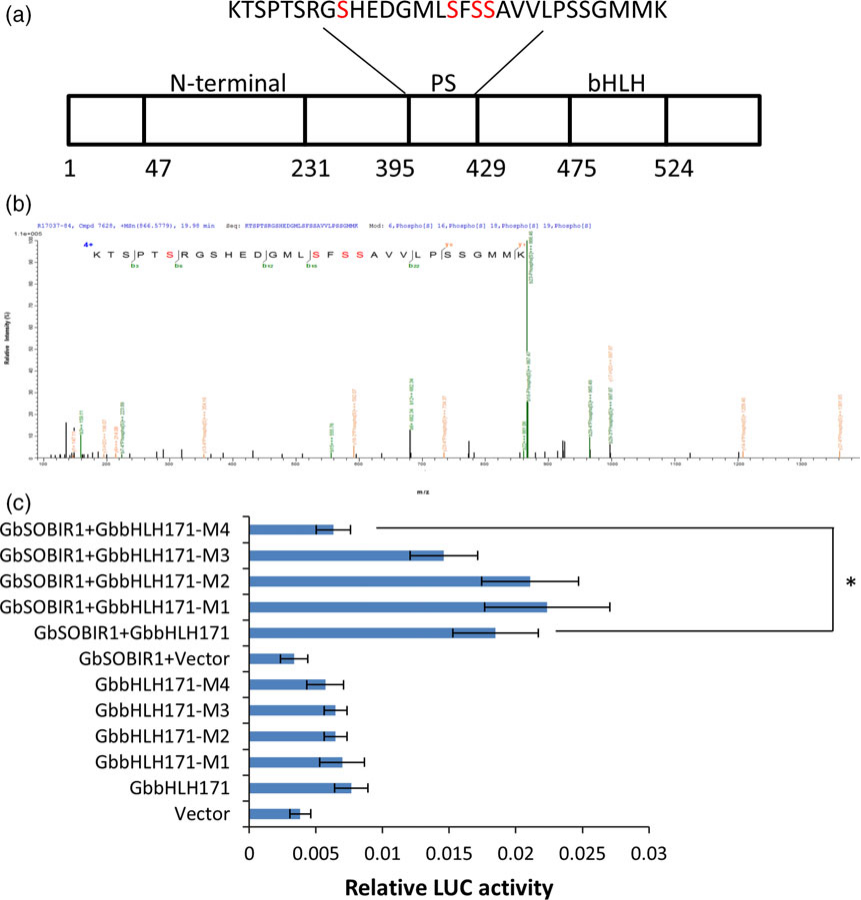
GbSOBIR1 phosphorylates GbbHLH171 at Ser413 site. (a) Schematic representation of GbbHLH171 structural domains. PS: predicted phosphorylation sites. bHLH: basic helix‐loop‐helix domain. (b) Identification of GbbHLH171 phosphorylation by mass spectrometry. Shown is a collision‐induced dissociation mass spectrum of the phosphopeptide KTSPTSRGSHEDGMLSFSSAVVLPSSGMMK. (c) LUC signal intensity involving different GbbHLH171 mutants. GbbHLH171‐M1 protein has Ser‐403 replaced with Ala, M2 has Ser‐410 replaced with Ala, M3 has Ser‐412 replaced with Ala, and M4 has Ser‐413 replaced with Ala. The values are the means ± SD, statistical analyses were performed using Student's *t* test (*P < 0.05). The experiments were repeated three times with similar results.

To identify the GbSOBIR1‐mediated phosphorylation site of GbbHLH171, the co‐immunoprecipitated proteins from tobacco leaves transiently expressing *GbSOBIR1* and *GbbHLH171* were subjected to mass spectrometric analysis, which revealed several sites with high probability (Figure 7a,b). To determine the physiological function of these potential phosphorylation sites and whether they are GbSOBIR1‐dependent, phosphorylation defective GbbHLH171 mutants carrying serine to alanine mutations (i.e. M1:bHLH171^S403A^, M2:bHLH171^S410A^, M3:bHLH171^S412A^, M4:bHLH171^S413A^) were examined in Luc reporter assays. As shown in Figure 7c, co‐expressing bHLH171^S413A^, GbSOBIR1 and GbJAZ2Pro:Luc resulted in lower fluorescence intensity than co‐expressing the original GbbHLH171, GbSOBIR1 and GbJAZ2Pro: Luc. The remaining GbbHLH171 mutants had similar results to the wild‐type protein, revealing that the S413A mutation, but not the other three mutations, affected the transcriptional activity of GbbHLH171. These results support the view that Ser413 is an *in vivo* phosphorylation site of GbbHLH171 by GbSOBIR1.

## Discussion

During plant–pathogen interactions, R proteins play an important role by perceiving pathogen effectors. While the majority of R proteins are cytoplasmic, many R proteins that play a role in plant *defence* against apoplastic fungal pathogens are receptor‐like proteins (RLPs) with transmembrane and extracellular LRR domains (Stotz *et al*., 2014). Subsequent to detecting the effectors, several other components, including receptor‐like kinases (RLKs), are required in order to transduce the signal downstream. Several RLKs play important roles in plant innate immunity. For example, FLAGELLIN‐SENSING 2 (FLS2) and CHITIN ELICITOR RECEPTOR KINASE 1 (CERK1) perceive bacterial flagellin and fungal chitin, respectively, as pathogen‐associated molecular patterns (PAMPs) (Chinchilla *et al*., 2007; Miya *et al*., 2007). These two RLKs are considered to be pattern recognition receptors (PRRs) and initiate PAMP‐triggered immunity (PTI) (Dodds and Rathjen, 2010). Members of the SOMATIC EMBRYOGENESIS RECEPTOR KINASE (SERK) family, particularly SERK3, also known as BRI1‐ASSOCIATED RECEPTOR KINASE 1 (BAK1), contribute to cell death and *defence* responses (Fradin *et al*., 2011; Roux *et al*., 2011). BAK1 interacts with various *defence*‐related proteins and acts as a versatile player in plant immunity (Chinchilla *et al*., 2009). BOTRYTIS‐INDUCED KINASE 1 (BIK1) of *Arabidopsis*, which is a receptor‐like cytoplasmic kinase (RLCK), has been identified as a critical component in the *defence* response against fungal necrotrophs such as *B. cinerea* and *A. brassicicola*. BIK1 acts in an MAPK cascade‐independent manner, and its kinase activity has been shown to be essential for its function as a regulator in *Arabidopsis* immunity (Lin *et al*., 2014; Mitsiades *et al*., 2006). Here, we identified another RLK, GbSOBIR1, as a positive regulator of cotton resistance against *V. dahliae*.

SOBIR1 is reported to be an important component in RLP‐initiated immunity. In tomato, SlSOBIR1 is required for Cf4‐ and Ve1‐mediated immunity against *C. fulvum* and *V. dahliae*, respectively (Liebrand *et al*., 2013). In addition, several RLPs from *A. thaliana* form a complex with AtSOBIR1. SOBIR1 binds to AtRLP23 and mediates NLP (necrosis and ethylene inducing peptide 1‐like protein)‐triggered immunity (Albert *et al*., 2015). AtRLP42, which was identified as RESPONSIVENESS TO BOTRYTIS POLYGALACTURONASES1 (RBPG1), was shown to bind AtSOBIR1 to confer fungal endopolygalacturonase‐induced resistance (Zhang *et al*., 2014). Another *Arabidopsis* immune receptor is RLP30, which perceives SCLEROTINIA CULTURE FILTRATE ELICITOR1 (SCFE1) from *Sclerotinia sclerotiorum* and is functionally dependent on SOBIR1 (Zhang *et al*., 2013). In this study, we cloned *GbSOBIR1* based on RNA‐Seq comparisons between lesion mimic mutant *ssn* and WT seedlings. VIGS was employed to knock down the expression of *GbSOBIR1*, and *TRV*:*GbSOBIR1* plants exhibited much more severe symptoms after *V. dahliae* inoculation compared to control plants, implying that GbSOBIR1 could be a key factor in cotton resistance to *V. dahliae*.

While SOBIR1 interacts with many effector‐perceiving RLPs, its downstream signalling network remains unclear except that it could form a complex with BAK1 in the presence of specific ligands or in the absence of BIR1 (Albert *et al*., 2015; Liu *et al*., 2016). In this study, we used the kinase domain of GbSOBIR1 to screen a Y2H library and identify an interacting protein—GbbHLH171, a *defence*‐related transcription factor. This interaction is further verified through immunoblot and BiFC assays. Another example of direct interaction of an RLK and a transcription factor is rice XA21 and OsWRKY62 (Peng *et al*., 2008). In this case, XA21 is cleaved to release the intracellular kinase domain and interacts with the OsWRKY62 transcriptional regulator exclusively in the nucleus of rice protoplasts (Park and Ronald, 2012). Our results demonstrate a different scenario in which the RLK GbSOBIR1 interacts with the transcription factor GbbHLH171 at the plasma membrane.

As an RLK, the kinase domain of SOBIR1 is essential for its function. Indeed, both the kinase and LRR domains of SOBIR1 are required for Cf4/Avr4‐induced HR, although they are dispensable for interaction with Cf4 (Bi *et al*., 2016). Until now, no phosphorylation substrates of SOBIR1 had been identified, and the mechanism by which its kinase activity facilitates resistance remained unknown. In this study, we demonstrate that GbSOBI1 phosphorylates GbbHLH171 at Ser413, which is essential for the transcriptional activity of GbbHLH171. This could be the first substrate identified for SOBIR1‐type kinases.

Although bHLH transcript factors are proven to be important regulators in various biological processes (Feller *et al*., 2011), how they are regulated is still unclear. Studies during the past few years have demonstrated that post‐translational modifications, particularly phosphorylation of bHLH transcript factors, affect their function. For example, phosphorylation at Thr328 of MYC2 is essential for its transcriptional activity and functional role in the JA response, but the kinase that is responsible for this phosphorylation is still unknown (Zhai *et al*., 2013). In another study, MYC2 was reported to be phosphorylated by MPK6 at a different site, Ser123, in a blue light‐dependent manner, and this phosphorylation is required for its function (Sethi *et al*., 2014), suggesting there are multiple sites for phosphorylation in one bHLH transcription factor. Furthermore, a rice bHLH transcription factor, OsRAI1, has been shown to be phosphorylated by OsMAPK3/6 and the active form of OsMKK4 *in vitro* (Kim *et al*., 2012). Here, we demonstrate a new kind of kinase in addition to the members in the MAPK cascade, GbSOBIR1, which can use a bHLH transcription factor as a substrate.

A role for SOBIR1 in development has also been described. Arabidopsis *nev* mutants show impaired floral organ shedding after flowering (Liljegren *et al*., 2009). A screen for mutations in *nev* plants that restore organ shedding identified a mutation in *SOBIR1* that resulted in premature floral organ shedding. Hence, the name EVERSHED (EVR) was coined as a synonym for this RLK, which in this case functioned as an inhibitor of abscission (Leslie *et al*., 2010). SOBIR1 was also found to interact with RLPs linked to development in tomato, including SlTMM and SlCLV2 (Liebrand *et al*., 2013). In this study, we failed to generate transgenic plants with *GbSOBIR1* overexpression or RNAi‐mediated knock‐down via *A. tumefaciens*‐mediated cotton transformation, due to abnormal development during embryogenesis. This suggests that the GbSOBIR1 expression level may affect the plant tissue culture process and that very low or very high expression levels may result in developmental abnormalities or autoimmunity, respectively, in this process.

We showed previously that GbJAZ2 could interact with GbbHLH171 and inhibit the transcriptional activity of GhbHLH171 (He *et al*., 2018). In this study, we demonstrated that GbSOBIR1 could phosphorylate GbbHLH171 and therefore facilitate its transcriptional activity. As a transcription factor, it seems that GbbHLH171 is under strict post‐translational regulation, both positively and negatively, suggesting it may be a core factor in cotton *defence*. This is similar with the case of MYC2, which is regulated by the JAZ complexes and kinases (Kazan and Manners, 2013; Sethi *et al*., 2014; Zhai *et al*., 2013). However, whether these two kinds of post‐translational regulation of GbbHLH171 are related and how exactly GbbHLH171 activated cotton resistance response require further study.

SOBIR1 forms complexes with RLPs, which are considered to be important pathogen‐perceiving R proteins during plant–fungus interactions. In cotton, several *defence*‐related RLPs have been identified based on sequence similarity of tomato *Ve1*, for example *GbVe1*,* Gbvdr5*,* Gbvdr6* (Yang *et al*., 2015, 2017; Zhang *et al*., 2012a). However, none of their corresponding PAMPs or effectors have been characterized and the molecular mechanism of how these RLPs are involved in *defence* remains unknown. Whether one or more of these RLPs could form complexes with GbSOBIR1 and activate *defence* response by perceiving certain PAMPs or effectors is worth studying in the future (Figure S5).

## Experimental procedures

### Plant materials, VIGS experiments and disease assays

Cotton (*Gossypium barbadense*) ‘7124’ and *Gossypium hirsutum* ‘YZ1’ plants were used in this study. For the VIGS experiments and disease assays, plants were grown in a growth room with a 16‐h‐day/8‐h‐night cycle at 25 °C.

The TRV vectors and Agrobacterium tumefaciens for VIGS were prepared according to Fradin *et al*. (2009). Inserts to generate *TRV: GbSOBIR1*,* TRV:GbbHLH171* were amplified from the cDNA of *G. barbadense* cv 7124. Primer pairs to generate TRV vectors are shown in supplemental Table S1. PCR fragments were cloned into the *TRV:00* plasmid using *Bam*HI and *Kpn*I. The constructs were transformed to *A. tumefaciens* GV3101 by electroporation. The vectors were agroinfiltrated into the cotyledons of 10‐d‐old cv 7124 plants using a needleless syringe as described previously (Gao *et al*., 2013a). Two weeks after infiltration, RNA was extracted from cotton roots to measure the expression of the target genes.

The *Verticillium dahliae* strain V991 was cultured on potato dextrose agar at 25 °C for 4 days and then further incubated on fresh potato dextrose agar medium for another 7 days. The conidia of *V. dahliae* were collected and resuspended in distilled water. The *V. dahliae* infection assays were performed by root dipping in spore suspension (2 × 10^5^ spores/mL) as described previously (Xu *et al*., 2011). Leaves of Inoculated plants were classified according to the disease symptoms, Lv0: healthy leaves, Lv1: leaves showing some necrosis or chlorosis, Lv2: leaves showing severe necrosis or chlorosis, Lv3: dead/shed leaves. The *in planta V. dahliae* biomass quantification in *Arabidopsis* and cotton was carried out according to (Fradin *et al*., 2011) and (Song *et al*., 2018), respectively.

### Gene cloning, vector construction and plant transformation

The full‐length sequence of *GbbHLH171* was identified in the *Gossypium barbadense* database (Yuan *et al*., 2015) and was obtained through PCR using *V. dahliae*‐infected cotton root cDNA as the template. The full‐length coding sequence of *GbbHLH171* was inserted into the binary plant vector pK2GW7.0 (Ghent University, http://www.plantgenetics.rug.ac.be/gateway/) to construct the vector *35S:GbbHLH171* for overexpression. The overexpression vector was introduced into *G. hirsutum* ‘YZ1’ plants by *A. tumefaciens* (strain EHA105)‐mediated transformation as described previously (Jin *et al*., 2006). The overexpression vector was transferred into *A. tumefaciens* strain GV3101 to transform the Arabidopsis (*Arabidopsis thaliana*) ecotype Columbia‐0 using the floral dip method (Clough and Bent, 1998).

### Nucleic acid extraction and expression analysis

Total RNA was isolated as described previously (Tan *et al*., 2013). For RT‐PCR and qRT‐PCR analyses, RNA was reverse‐transcribed to cDNA using SuperScript III reverse transcriptase (Invitrogen). qRT‐PCR was performed using the ABI Prism 7000 system (Applied Biosystems, CA). The values are given relative to the housekeeping genes *UB7* in cotton. The primers that were used in the RT‐PCR and qRT‐PCR are listed in Supplemental Table S1.

### Yeast‐two‐hybrid assays

The *V. dahliae*‐inoculated cotton root library for Y2H screening was constructed with the Matchmaker Gold Yeast‐Two‐Hybrid System (Clontech, Cat. no. 630489). The kinase domain of the cotton *GbSOBIR1* gene was fused with the GAL4 DNA‐binding domain in pGBKT7 to ensure that there was no autoactivation and toxicity due to the X‐a‐Gal assay in yeast; the GbSOBIR1 fusion protein was used as bait to identify interacting proteins. To detect protein–protein interactions between GbSOBIR1 and the identified proteins, the full‐length *GbbHLH171* genes were cloned into pGADT7. pGBKT7 fused with GbSOBIR1 was used to transform the Y2H yeast strain, and pGADT7 fused with GbbHLH171 was transferred into the Y187 yeast strain using the Transformation System (Clontech, Cat. no. 630489). Interactions between these proteins after mating were determined by growth on SD medium with the ‐Trp/‐Leu/‐His/‐Ade/‐X‐α‐Gal assay as described by the manual (Clontech, Cat. no. 630489).

### Co‐immunoprecipitation

The *Agrobacterium tumefaciens* GV3101 strains containing pGWB417‐35s‐GbSOBIR1‐Myc, pGWB415‐35s‐GbbHLH171‐HA or an empty pGWB417‐35s‐Myc vector were grown in fresh LB medium supplemented with appropriate antibiotics and grown to the optical density of OD600 = 1. Cultures were pelleted and resuspended in buffer containing 10 mm MES (pH 5.6) and 150 μm acetosyringone. Agrobacteria carrying different constructs were mixed 1:1 (OD600 = 0.8) and infiltrated into leaves of 4‐week‐old *N. benthamiana* plants. Samples were harvested 60 h later, and 5 g of tissue was ground in liquid nitrogen and resuspended in 10 mL volume of grinding buffer (50 mm Tris‐HCl 7.5, 150 mm NaCl, 1 mm EDTA, 1% NP‐40, one tablet of protease inhibitor mixture; Roche). The extracted proteins were subjected to co‐immunoprecipitation using a Pierce magnetic c‐Myc‐tag IP/Co‐IP kit (Thermo Scientific, Cat. no. 88844). The eluted proteins were subsequently analysed by Western blot using anti‐Myc or anti‐HA antibodies.

### BiFC analysis

For bimolecular fluorescence complementation (BiFC) analysis experiments, the full‐length coding region of GbSOBIR1 was cloned into pS1301nYFP, and the GbbHLH171 sequence was cloned into pS1301cYFP. The fusion constructs were transformed into *A. tumefaciens* strain GV3101. Then, the positive‐transformed cells were cultured at 28 °C in LB medium with kanamycin (50 μm) and rifampicin (50 μm). The cell cultures were then infiltrated into tobacco leaves as described above. The interactions were detected under fluorescence light using a Leica TCS SP2 confocal spectral microsystems laser‐scanning microscope (Leica, Heidelberg, Germany).

### *In vitro* phosphorylation assays

Cell‐free protein expression and *in vitro* kinase assays were performed as previously reported (Min *et al*., 2015). The cell‐free‐expressed kinase GbSOBIR1 and the *E. coli*‐induced and ‐expressed substrate GbbHLH171‐GST were mixed with kinase assay buffer (40 mm HEPES, pH 7.5, 130 mm KCl, 10 mm MgCl_2_, 0.1 mm ATP, 5 mm dithiothreitol, 5 mm b‐glycerophosphate and 0.2 mm sodium orthovanadate). The reactions were incubated at 37 °C for 15 min and then stopped by boiling for 5 min. The phosphorylation assay products were separated by electrophoresis using Phos‐tag SDS‐PAGE (8% acrylamide gels, 0.375 m Tris, 0.1% SDS, 0.1 mm MnCl_2_ and 0.05 mm Phos‐tag AAL‐107), and the gels were stained with Coomassie Brilliant Blue, destained and visualized.

### Dual‐luciferase reporter assays

The transient dual‐luciferase reporter assays were performed as described previously (Hellens *et al*., 2005). The promoter sequence of *GbJAZ2* was amplified by PCR using cv7124 genomic DNA and was cloned into pGreenII 0800‐LUC at the *Pst*I and *Bam*HI sites. The coding regions of *GbbHLH171* and *GbSOBIR1* were amplified by PCR and cloned into pGreenII 62‐SK at the *Pst*I and *Bam*HI sites to generate *35::GbbHLH171* and *35::GbSOBIR1*. For the mutated version of GbbHLH171, different primers were used for site‐directed mutagenesis by overlap extension PCR. The pGreenII 62‐SK clone harbouring the wild‐type GbbHLH171 insert was used as a template for overlap extension PCR. The PCR product was then cloned into pGreenII 62‐SK as well. Leaves of 4‐week‐old *N. benthamiana* plants were infiltrated with *A. tumefaciens* carrying different vectors. Sixty hour later, these leaves were collected and were ground in liquid nitrogen. Firefly luciferase and *Renilla* spp. luciferase activities were quantified using the dual‐luciferase assay reagents (Promega, Madison, WI) with a multimode plate reader (PerkinElmer, Waltham, MA).

### Identification of phosphorylation site

Purified protein from the Co‐IP experiment was digested according to the FASP procedure described previously (Wisniewski *et al*., 2009). LC‐MS/MS experiments were performed on a quadrupole‐Orbitrap‐liner ion trap hybrid mass spectrometer system (Orbitrap Fusion Lumos) that was coupled to Ultimate 3000 RSLCnano system (Thermo Fisher Scientific, Germany). The peptide mixture (2 μg) was loaded onto an Acclaim PepMap 100 analytical column (75 μm × 15 cm, C18, 3 μm, Thermo Scientific) in buffer A (0.1% formic acid) and separated with a linear gradient of buffer B (80% acetonitrile and 0.1% formic acid) at a flow rate of 300 nl/min over 60 min. Data‐dependent acquisition with full scans in the 350–1500 m/z range was carried out using an Orbitrap mass analyser at a mass resolution of 60 000 at 200 m/z. Most intense precursor ions were selected using the top speed data‐dependent mode with a maximum cycle time of 3 s. Peptides with charge 2–7 were selected, and dynamic exclusion time was 40 s. Precursor ions were fragmented using higher‐energy collision dissociation (HCD), and MS/MS ions were detected using Orbitrap at a mass resolution of 30 000 at 200 m/z. For data analysis, MS/MS spectra were searched using MASCOT engine (Matrix Science, London, UK) embedded into Proteome Discoverer 2.1 (Thermo Electron, San Jose, CA).

## Conflict of interest

The authors declare no conflict of interest.

## Supporting information


**Figure S1** Amino acid sequence alignment of GbSOBIR1, SlSOBIR1, AtSOBIR1 and NbSOBIR1.
**Figure S2** *GbSOBIR1* expression level after exogenous treatment with defense‐related phytohormones.
**Figure S3** Expression profile of *GbbHLH171*.
**Figure S4** Sequence analysis of *GhJAZ2* promoter. G‐box regions are highlighted in cyan.
**Figure S5** Schematic model of how GbSOBIR1 and GbbHLH171 regulate defense response in cotton plant.
**Table S1** List of primers used in this study.
