# Supplementary material for: GbSOBIR1 confers Verticillium wilt resistance by phosphorylating the transcriptional factor GbbHLH171 in Gossypium barbadense
Source: Plant Biotechnol J. 2018 Jul 24;17(1):152–63. doi: 10.1111/pbi.12954 (PMC6330551; doi:10.1111/pbi.12954)
Supplement: Supplementary file 1 — Figure S1 Amino acid sequence alignment of GbSOBIR1, SlSOBIR1, AtSOBIR1 and NbSOBIR1. Figure S2 GbSOBIR1 expression level after exogenous treatment with defense‐related phytohormones. Figure S3 Expression profile of GbbHLH171. Figure S4 Sequence analysis of GhJAZ2 promoter. G‐box regions are highlighted in cyan. Figure S5 Schematic model of how GbSOBIR1 and GbbHLH171 regulate defense response in cotton plant. Table S1 List of primers used in this study. [file PBI-17-152-s001.pdf]

Table S1 List of primers used in this study.

Primer code	Sequence,5'-3'	Target sequence
UB7-S	GAAGGCATTCCACCTGACCAAC	GbUB7 qRT-PCR
UB7-A	CTTGACCTTCTTCTTCTTGTGCTTG	GbUB7 qRT-PCR
GhbHLH171-S	ATTTCCCACCATCCCTACAT	GhbHLH171
GhbHLH171-A	CACTTCCGATTTTGGATATTAG	GhbHLH171
GhbHLH171-RT-S	GCTCCCCAATCTCACCCCTC	GhbHLH171 RT-PCR
GhbHLH171-RT-A	CAAAAACCTGCCCTTGCCTC	GhbHLH171 RT-PCR
GhbHLH171-TRV-S	AACCCCAAACGCAACCTCAA	GhbHLH171 VIGS fragment
GhbHLH171-TRV-A	TGGAGACGCTGCTGCTGGAGAGT	GhbHLH171 VIGS fragment
171-M1-S	CACTTCCAGAGGGGCTCATGAAGATGG	GhbHLH171 mutation
171-M1-A	CCATCTTCATGAGCCCCTCTGGAAGTG	GhbHLH171 mutation
171-M2-S	GATGGGATGCTTGCCTTTTCTTCTGCTG	GhbHLH171 mutation
171-M2-A	CAGCAGAAGAAAAGGCAAGCATCCCATC	GhbHLH171 mutation
171-M3-S	GATGGGATGCTTTCCTTTGCTTCTGCTG	GhbHLH171 mutation
171-M3-A	CAGCAGAAGCAAAGGAAAGCATCCCATC	GhbHLH171 mutation
171-M4-S	GATGGGATGCTTTCCTTTTCTGCTGCTG	GhbHLH171 mutation
171-M4-A	CAGCAGCAGAAAAGGAAAGCATCCCATC	GhbHLH171 mutation
27real-F	TACCATCACCTAAACACAAACACAAA	GbSOBIR1 qRT-PCR
27real-R	CACTGAGAAGACAAACCCAGATAAAC	GbSOBIR1 qRT-PCR
27KD-F	TTGGATTCACTAGAGATCATAGGCA	GbSOBIR1 Kinase domain
27KD-R	TCGCTCTTGATTTGAGACAACATG	GbSOBIR1 Kinase domain
GbSOBIR1-F	CAAAGGCCCAGTCAACACTAACCAT	GbSOBIR1
GbSOBIR1-R	ATTATTGGTTTGACCTTCGCTCTTG	GbSOBIR1
GbSOBIR1-RT-F	GAGGCAACAAGCAATAATGGAGGT	GbSOBIR1 RT-PCR
GbSOBIR1-RT-R	CTTTCTCTAGAAATGCTAAATCTTCAGC	GbSOBIR1 RT-PCR
GbSOBIR1-V-F	CGGGGTACCTACCATCACCTAAACACAAACACAAA	GbSOBIR1 VIGS fragment
GbSOBIR1-V-R	CGCGGATCCCACTGAGAAGACAAACCCAGATAAAC	GbSOBIR1 VIGS fragment
GbJAZ2-pro-F	CAAGAAGGAAAATCTTATAGGAAGG	GbJAZ2 promoter
GbJAZ2-pro-R	AACTTTGAACATCACGAATCTG	GbJAZ2 promoter
ITS1-F	AAAGTTTTAATGGTTCGCTAAGA	*V.dahliae*
ST-VE1-R	CTTGGTCATTTAGAGGAAGTAA	*V.dahliae*

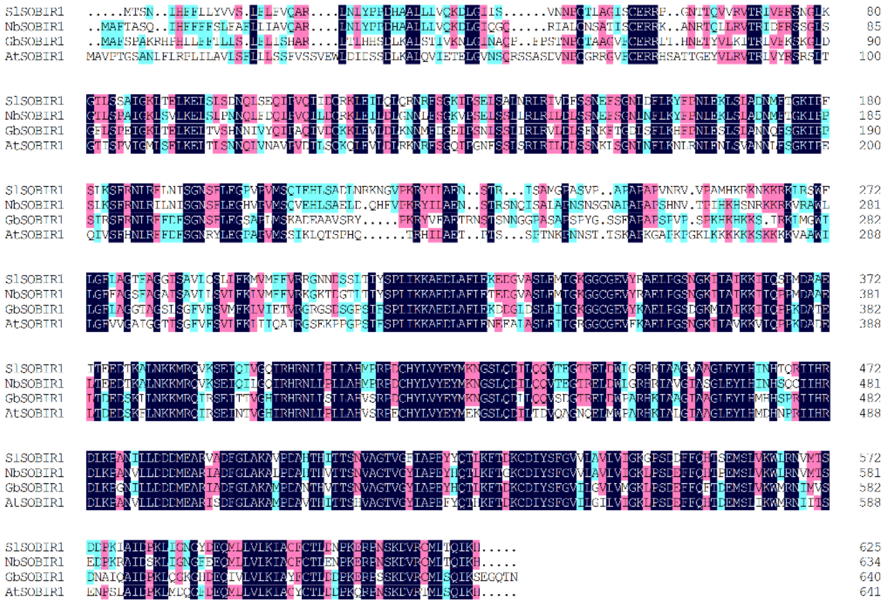

Fig. S1 Amino acid sequence alignment of GbSOBIR1, SlSOBIR1, AtSOBIR1 and NbSOBIR1. Amino acid residues highlighted in dark blue and purple represent identical residues in all four or three of the aligned protein sequences, respectively. Residues in light blue or white represent residues present in two sequences or are unique residues, respectively.

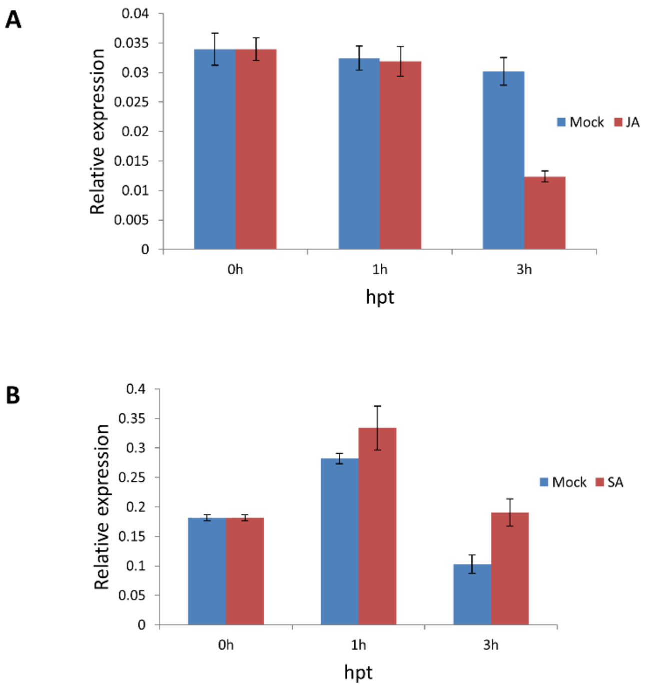

Fig. S2 *GbSOBIR1* expression level after exogenous treatment with defense-related phytohormones.
(A) Expression levels of *GbSOBIR1* after treatment of JA.
(B) Expression levels of *GbSOBIR1* after treatment of SA.
The plants were pretreated with Hoagland's solution. For treatments with MeJA and SA, 0.1 mM and 1 mM concentrations in Hoagland solution were used, respectively. The roots were harvested at different time points after treatment for subsequent RNA isolation. Four plants were harvested for each time point and treatment. The values are the means ± SD for three technical replicates. The transcript levels of each gene were normalized to *UB7*. hpt, hours post treatment.

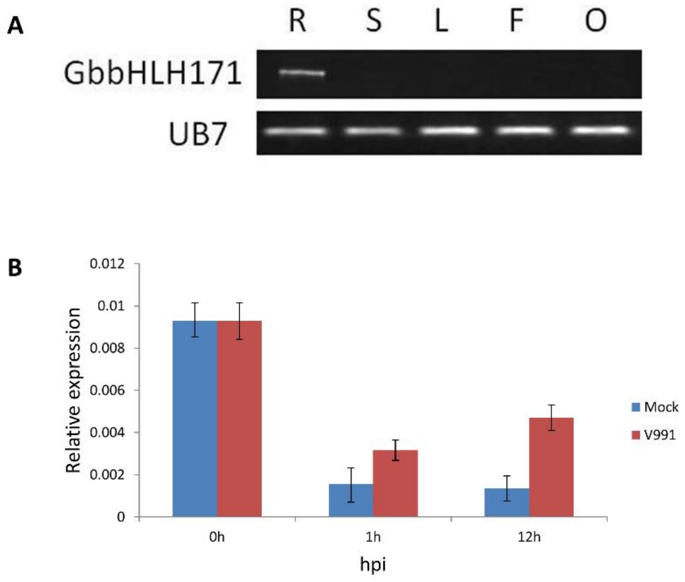

Fig. S3 Expression profile of *GbbHLH171*.
(A) RT-PCR analysis of *GbbHLH171* expression in different tissues. Total RNA was isolated from roots (R), stems (S), leaves (L), flower (F), anther (A) and ovule (O) of the WT cotton line 7124. The *UB7* gene was used as a control.
(B) qRT–PCR analysis of *GbbHLH171* expression in cotton roots inoculated with *V. dahliae*. The values are the means ± SD for three technical replicates. The transcript levels of *GbbHLH171* were normalized to *UB7*. hpi, hours post inoculation.

```
GbJAZ2 prompter sequence (1499bp)
+ TTGAATAAAT TATATCTAAA GTCATTGAAT TATTAGTAAG TTTACATTTT ACTTATTCAA CTTTAAAATG
- AACTTATTTA ATATAGATTT CAGTAACTTA ATAATCATTC AAATGTAAAA TGAATAAGTT GAAATTTTAC

+ TTACGAAATG GTCGCTGAAC TATTCGAAAA TTTTCATTCA AGTCACTAGA CTATTTGAAA GTTTTATTTA
- AATGCTTTAC CAGCGACTTG ATAAGCTTTT AAAAGTAAGT TCAGTGATCT GATAAACTTT CAAAATAAAT

+ AGTTGCTAGA TTGTTAATTT TTTTTAAAGT TCGGCTAATG AGCTCTAAGC GATGATTCGA CAATTAGTAC
- TCAACGATCT AACAATTAAA AAAAATTTCA AGCCGATTAC TCGAGATTCG CTACTAAGCT GTTAATCATG

+ TCTTTGAAGA ATGGAAGAAT ATACCTTAGA TATAAATTGA TCTAACGGTC AGTGTCGAAG ATTGGAGCAA
- AGAAACTTCT TACCTTCTTA TATGGAATCT ATATTTAACT AGATTGCCAG TCACAGCTTC TAACCTCGTT

+ AAAACTGTTA GGACTTTAGG TCACAGATTC GTGATGTTCA AAGTTATTCT ATGAAAAATA ACTATACTAT
- TTTTGACAAT CCTGAAATCC AGTGTCTAAG CACTACAAGT TTCAATAAGA TACTTTTTAT TGATATGATA

+ AGAAAAGATT GGGAAAGAAA GCTTTCAATT AGTGTAAATG GTATGAATAA AGAAAGCTAT ACAACAATGA
- TCTTTTCTAA CCCTTTCTTT CGAAAGTTAA TCACATTTAC CATACTTATT TCTTTCGATA TGTTGTTACT

+ TTTTAACATC TTGGTGACTA AAATGAACTT TTAAATAATC TAATGACCAT TTTGTAATTA TTTTAAAATT
- AAAATTGTAG AACCACTGAT TTTACTTGAA AATTTATTAG ATTACTGGTA AAACATTAAT AAAATTTTAA

+ AAGTGCTCAA AACTTAAATA TACTAATCCT AAATTAATTA AATAAGAATA AAAATGGTGT AGGGATATTT
- TTCACGAGTT TTGAATTTAT ATGATTAGGA TTTAATTAAT TTATTCTTAT TTTTACCACA TCCCTATAAA

+ GGCGCAGGAG GCATCCAAAG TGTGCAGGCT CCTATTGGGG AAAGGGGCAT GTGGATTGGC ACATGTGCAA
- CCGCGTCCTC CGTAGGTTTC ACACGTCCGA GGATAACCCC TTTCCCCGTA CACCTAACCG TGTACACGTT

+ TTATTATTGG AGAAAGCAAC GTGCGGTTGC CTCTTTTGAG ATTCAAGGAA GGCAAGAGAA GAGAAGGCGG
- AATAATAACC TCTTTCGTTG CACGCCAACG GAGAAAACTC TAAGTTCCTT CCGTTCTCTT CTCTTCCGCC

+ TGTTTGTGGC GCCCACACAA AATGAGGATC ATGCACTCAT TTTAAATTTT TATATTTAAG CTGACTTGTT
- ACAAACACCG CGGGTGTGTT TTACTCCTAG TACGTGAGTA AAATTTAAAA ATATAAATTC GACTGAACAA

+ TTACAAAACT ACCCTTTTTT CACTAATGAA CTAATGAACT TTTGATCAAA ATCATAAAGC TCAAAATATT
- AATGTTTTGA TGGGAAAAAA GTGATTACTT GATTACTTGA AAACTAGTTT TAGTATTTCG AGTTTTATAA

+ CTTATTTGTA GAAAAAATCT AAAATGAGAG GAAAAAGGAA GGAAAATATA TTATAAATAT ATAAATAATA
- GAATAAACAT CTTTTTTAGA TTTTACTCTC CTTTTTCCTT CCTTTTATAT AATATTTATA TATTTATTAT

+ ATATTTTGTC ATACCACCAT TTTTAATTAA GGTCAAAGAG AAAAAAAAGG AAAACTTAGA CACCAGTTAT
- TATAAAACAG TATGGTGGTA AAAATTAATT CCAGTTTCTC TTTTTTTTCC TTTTGAATCT GTGGTCAATA

+ GAAAAAAAAA TTATTTTATT TTCAACCTCA TCTCGAAGAC TAATTGAGTT TTGCTATCAA AGTTGGTATG
- CTTTTTTTTT AATAAAATAA AAGTTGGAGT AGAGCTTCTG ATTAACTCAA AACGATAGTT TCAACCATAC

+ AAATTTATAA TTTGCAACTT TGTAATTCCT AGTGTTTAAT CAAACAAAAA AGAAATAGTA ATTGCCCCAT
- TTTAAATATT AAACGTTGAA ACATTAAGGA TCACAAATTA GTTTGTTTTT TCTTTATCAT TAACGGGGTA

+ AATTTAATTA AATCCCTATA ATCAAGGAAT TTCGAAGATA CTTTTACCAA TATTAAAATA TAAAAAAATA
- TTAAATTAAT TTAGGGATAT TAGTTCCTTA AAGCTTCTAT GAAAATGGTT ATAATTTTAT ATTTTTTTAT

+ AACTGATAAA ACATCGCCGT CGACACAGAT ACAGAAAAGA AAAAACAAAA GAACACGTGT TGAAAATCCC
- TTGACTATTT TGTAGCGGCA GCTGTGTCTA TGTCTTTTCT TTTTTGTTTT CTTGTGCACA ACTTTTAGGG

+ AATCAAAGGA GACGAGACGA TACAATAGTG AAGCGCCCTC ACGAGTTACG AACCTGTAGG AGCTTAAAAA
- TTAGTTTCCT CTGCTCTGCT ATGTTATCAC TTCGCGGGAG TGCTCAATGC TTGGACATCC TCGAATTTTT

+ AAAGCCTCAT TTATTCCTCG TGCTTTTTAA CCCACCTTCC TCTACCACCT TTGTTTTTTT CTAAGATTAA
- TTTCGGAGTA AATAAGGAGC ACGAAAAATT GGGTGGAAGG AGATGGTGGA AACAAAAAAA GATTCTAATT

+ AAACATAAAT AAAATCTCTT TGCTTAGCCT AACATCATAT TTTTCGAACA GATCCTTCGT TTAGCTGTAA
- TTTGTATTTA TTTTAGAGAA ACGAATCGGA TTGTAGTATA AAAAGCTTGT CTAGGAAGCA AATCGACATT

+ GTAAAGTTTT AAAGATCATT CTCTGAGAA
- CATTTCAAAA TTTCTAGTAA GAGACTCTT

                                                          G-BOX
```

Fig. S4 Sequence analysis of *GhJAZ2* promoter. G-box regions are highlighted in cyan.

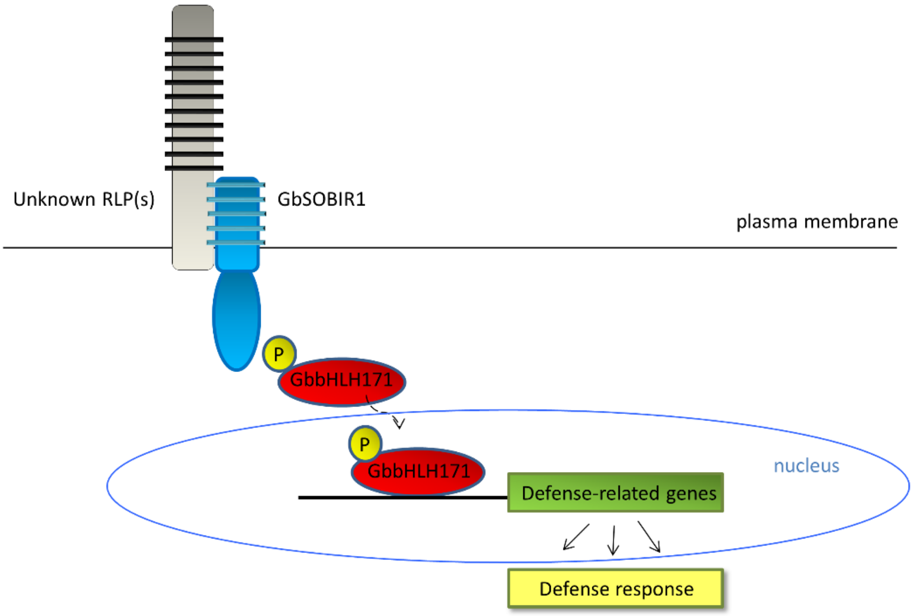

Fig. S5 Schematic model of how GbSOBIR1 and GbbHLH171 regulate defense response in cotton plant. GbSOBIR1 is predicted to form complexes with unknown RLP(s) on the plasma membrane. GbbHLH171 interacts with and is phosphorylated by GbSOBIR1 near the plasma membrane. The phosphorylated GbbHLH171 may enter the nucleus and bind to the promoter of defense-related gene(s) to activate their transcription, leading to defense responses in the cotton plant.
